# The Slippery Role of Induction Chemotherapy in Head and Neck Cancer: Myth and Reality

**DOI:** 10.3389/fonc.2020.00007

**Published:** 2020-01-23

**Authors:** Daris Ferrari, Maria Grazia Ghi, Ciro Franzese, Carla Codecà, Max Gau, Jerome Fayette

**Affiliations:** ^1^Department of Oncology, San Paolo Hospital, Milan, Italy; ^2^Oncology Unit 2, Istituto Oncologico Veneto-IRCCS, Padua, Italy; ^3^Department of Radiotherapy and Radiosurgery, Humanitas Cancer Center, Rozzano, Italy; ^4^Rhône-Alpes, Centre de Lutte Contre le Cancer Léon Bérard, Lyon, France

**Keywords:** induction, chemotherapy, chemoradiotherapy, radiotherapy, head and neck, squamous cell carcinoma

## Abstract

Chemoradiotherapy as an alternative to surgery can be offered to patients affected by loco-regionally advanced head and neck cancer (HNC). Induction chemotherapy is a valid option, supported by few positive trials, but its real efficacy is still a matter of debate. The standard regimen for induction chemotherapy in Europe is a combination of docetaxel (75 mg/m^2^) and reduced dose doses of cisplatin (75 mg/m^2^) and 5-fluorouracil (750 mg/m^2^ day, for five consecutive days) (TPF). It is less toxic and more effective than the historical therapy PF (cisplatin 100 mg/m^2^ and fluorouracil 1,000 mg/m^2^/day for five consecutive days). However, in some studies treatment-related mortality has been reported to be as high as 6%. Therefore, some less toxic combinations, such as a modified TPF regimen and the combination of carboplatin plus paclitaxel have been studied. These regimens are showing promising results but deserve further validation in comparative trials. Furthermore, several trials are underway in order to enhance TPF with immune checkpoints inhibitors. Compared to chemoradiotherapy, induction chemotherapy followed by chemoradiation was shown to be non-inferior, and it could decrease the distant metastatic progression, especially in high-risk populations. For selected patients, induction chemotherapy could be a strong option. The chemoselective process that leads to immediate surgery for non-responders, the high response rate (complete responses are sometimes observed), and the survival data, are all arguments in favor of induction chemotherapy, if performed in experienced centers involving health professionals in the context of a skilled multidisciplinary team.

## Introduction

Every year, over 650,000 head and neck cancer (HNC) cases are diagnosed, and they account for more than 330,000 deaths worldwide (1). Risk factors associated with HNC include tobacco use, alcohol consumption, and human papillomavirus infection.

Loco-regionally advanced head and neck squamous cell carcinomas (LAHNSCC) is the current presentation in more than half of the cases and different treatment approaches can be considered.

Depending on the site and the stage of the tumor, surgery, radiation therapy or chemoradiotherapy (CRT) are validated options. The recommended choice in squamous cell carcinomas of the oral cavity is surgery but, in other sites, alternative strategies are possible. When the aim is the preservation of organ function, or when surgery is unfeasible, an effective treatment consists of the combination of chemotherapy (CHT) and radiotherapy (RTX), either in a sequential or concomitant modality.

After many years of well-conducted and convincing trials, concomitant CRT is now regarded as the standard of care for fit patients affected by LAHNSCC. The best agent to be employed together with RTX is cisplatin, but its widespread use is limited by the frequent and severe toxicities. Current recommendations do not suggest cisplatin for patients with ECOG Performance Status (PS) > 1, renal failure, neurologic abnormalities, audiometric impairment, hepatic, and cardiovascular disease. For these patients, alternative concurrent regimens including cetuximab or carboplatin and 5-fluorouracil (5-FU) should be considered (2).

One still unresolved question refers to the best timing for the administration of CHT. The MACH-NC meta-analysis by Pignon et al. well-documented the superiority of CRT over RTX in terms of overall survival (OS). Whereas, the concomitant approach demonstrated to be the most effective one, it is not so evident how useful can be the delivering of CHT before radiation. This modality, defined as induction or neoadjuvant chemotherapy (IC), has been considered an intriguing and smart option for many reasons but, after years of studies and debates, its role has not been fully established.

The main objectives of IC can be summarized as follows:

- The reduction rate of loco-regional relapse and distant metastases.
- The selection of chemosensitive patients in order to reduce the intensity of subsequent RTX or CRT.
- Tumor shrinkage and organ preservation.
- Early treatment while waiting for the beginning of RTX (a real problem for many centers).

The main trials comparing IC followed by RTX alone or by concomitant CRT will be analyzed, with a special focus on the change of gear coming from the European trial TAX 323/EORTC 24971 and the American trial TAX 324, both published in 2007 (3–5). The first study delivered four cycles of TPF (docetaxel, cisplatin, 5-FU) vs. PF (cisplatin, 5-FU) followed by RTX and demonstrated significant survival benefits for TPF in a population of patients with previously untreated, unresectable LASCCHN (3). The second trial delivered 3 cycles of TPF vs. PF followed by concomitant carboplatin during RTX, in a population of patients either unresectable and of low surgical curability, or candidates for organ preservation. Again, the median OS was significantly higher in the TPF arm, that is now considered the standard treatment when the choice falls on IC (4, 5).

New options of radiation therapy have rapidly progressed in the recent years and now Intensity Modulated Radiotherapy (IMRT) is the preferred technique for its ability of better surrounding the tumor volume and the reduced toxicity compared to standard RTX.

An argument against IC is the burden of toxicity which is going to add up, whenever any CHT is combined with RTX. This issue will be analyzed taking into account the most recent trials and underlining the need to offer these complex treatments at high-volume centers, where the skills of the personnel involved are at the highest levels.

In summary, for unresectable non-laryngeal diseases, concomitant CRT is the standard, but IC prior to surgery or RTX (± concurrent CHT) remains an option. For laryngeal and hypopharyngeal cancer IC is strongly suggested, with the aim of maximizing organ preservation without compromising survival. In this review, we will focus on the different regimen of IC, the main modalities of radiation therapy and the toxicity associated with CHT and RTX. Then we will discuss on the controversial role of IC for unresectable non-laryngeal and laryngeal cancers, and the perspective for the future with the possible introduction of immunotherapy.

## Type and Dose of Radiotherapy

RTX details have not been extensively explained in the majority of the studies investigating the role of IC in LAHNSCC, above all in terms of the used technique. In one of the first trials published by Posner et al. (4) patients received concomitant CRT, with a radical dose ranging from 70 to 74 Gy, administered with conventional 2 Gy per fractions in 5 days per week. The dose administered to uninvolved lymph nodes was at least 50 Gy, while involved lymph nodes received among 60 and 74 Gy. No information regarding RTX technique was reported. Patients included in the trial by Vermorken et al. (3) were treated either with conventional fractionation, accelerated or hyperfractionated regimens. In the conventional group a total dose of 66–70 Gy was used, while in accelerated and hyperfractionated regimens a dose of 70 and 74 Gy was adopted, respectively. Patients were treated between 1999 and 2003, when IMRT was not yet widely diffused. In the subsequent trial published by Argiris et al. (6), patients were treated with cetuximab-containing induction regimen and then IMRT. Treatment volumes encompassed initially the primary tumor and nodal regions at risk to 50 Gy, then high-risk nodal regions received 60 Gy and the gross disease a final dose of 70 Gy, delivered in 35 fractions. At discretion of the radiation oncologist two patients received a boost to 72 and 74 Gy. The TREMPLIN trial included a conventional external beam radiation treatment with a radical dose of 70 Gy delivered in 2 Gy per fraction, however a percentage of patients interrupted RTX early, due to disease progression or toxicity (7). The RTOG trial, published in 2003 and followed by long-term results in 2013, provided for all patients a conventional dose of 70 Gy in 35 fractions (8, 9). The PARADIGM trial adopted different RTX schedule for treatment groups. For IC followed by CRT in the docetaxel group and CRT in the cisplatin only group, accelerated concomitant boost over 6 weeks was given, with a total dose of 72 Gy in 1.8/1.5 Gy fractions. For IC followed by chemoradiation within carboplatin group, RTX was administered once daily with a total dose of 70 Gy in 2.0 Gy fractions (10). Standard fractionation was adopted by other trials (11) and included mixed technique (12, 13). In the recently published GORTEC 2007-02 phase III randomized trial, a conventional dose of 70 Gy was adopted and IMRT technique was recommended, but 3D conformal RTX was also accepted (14). A similar approach was adopted in the trial by Chung et al. (15).

Considering the fast evolution of RTX technique in the last few decades (from 3DCRT to IMRT and Volumetric Modulated Arc Therapy), the heterogeneity between the most relevant studies, often within single trials, could bias the results both in terms of efficacy and tolerance of sequential CRT. In the future, all the trials on this setting should be designed in such a way as to have homogeneity both in terms of RTX technique, and also in terms of dose and fractionation (conventional vs. accelerated vs. hyperfractionated).

## The Choice of Induction Chemotherapy

### The Standard Regimen Is TPF

For fifty years, IC has been proposed for LAHNSCC. Several regimens were used, mainly platinum-based polychemotherapy.

The MACH-NC meta-analysis, updated in 2009 (16), with 93 trials and 17,346 patients, showed a significant advantage in OS for IC when cisplatin and 5-FU were used before RTX. Without consensus, doses and schemes of administration remained institution dependent.

The combination of cisplatin and 5-FU was confirmed by a comparative phase III trial of 237 patients with operable and inoperable disease (17). Patients were randomized in two arms: IC with cisplatin (100 mg/m^2^) and 5-FU (1,000 mg/m^2^/days for five consecutive days) for four cycles 3 weeks apart (PF) followed by RTX or RTX alone. There was a significant benefit in the OS rate at 5 and 10 years in the subgroup of inoperable patients (21 and 16% with induction vs. 8 and 6% without induction, *p* = 0.04).

In 2007, two trials showed a significant benefit of adding taxans. The TAX 324 trial (4) with 501 patients compared induction with PF to TPF (three cycles of 75 mg/m^2^ docetaxel, 100 mg/m^2^ cisplatin, both on day 1 and 1,000 mg/m^2^/day 5-FU by continuous intravenous infusion day 1–4), followed by concomitant chemoradiation with concurrent weekly carboplatin. With the addition of taxans to the PF combination, the OS rate went up to 71 months vs. 30 months with PF (*p* = 0.006). Moreover, the triple agent combination was well-tolerated, except for grade 3 and 4 neutropenia, which were more frequent in the TPF arm (83% vs. 56%). In total, fewer patients had treatment delays with the TPF regimen (29% vs. 65% for the PF scheme). The TAX 323/EORTC 24971 trial (3) performed an essay with lower doses of 5-FU (750 mg/m^2^/day by continuous intravenous infusion for five consecutive days, without bolus) and cisplatin (75 mg/m^2^ on day 1). The control arm consisted of cisplatin at the dose of 100/m^2^, administered on day 1, followed by 5-FU at the dose of 1,000 mg/m^2^/day, administered by continuous intravenous infusion for five consecutive days. Three hundred fifty-eight patients were randomly assigned, and median OS was higher in the TPF arm (18.8 months vs. 14.5 months, *p* = 0.02). Tolerance was also better for TPF, with 24.3% of patients not able to complete the treatment in the TPF arm vs. 35% in the PF arm. Mortality from IC was lower in the TPF arm, with 2.3% treatment-related deaths vs. 5.5% in the PF arm. In other phase III trials, mortality of TPF ranged from 2% to 7%. As in the TAX 324 trial, taxans addition increased the risk of neutropenia.

The most recent MACH-NC meta-analysis of five randomized trials (1,772 patients) focused on IC comparing PF with taxans (docetaxel or paclitaxel), cisplatin, and 5-FU (Tax-PF) supported those results, with a hazard ratio (HR) of death of 0.72 (95% CI, 0.63–0.83), and an absolute benefit in OS at 5 years of 7.4% in favor of Tax-PF (18). Tax-PF was also associated with better compliance, and only neutropenia and thrombocytopenia were more frequently reported with the Tax-PF regimen. The limitation of this analysis is related to the heterogeneity of the trials and some missing data. Indeed, different schemes were used for IC. Patient's and tumor's characteristics were also heterogeneous between the five trials.

In addition, quality of life was improved with TPF vs. PF in the TAX 323 trial (19) and the cost-utility analysis of both the TAX 323 and 324 studies showed benefit in quality-adjusted life-years in favor of TPF (20).

In conclusion, when IC has to be chosen, the strongest supported regimen is TPF.

### Less Toxic Schemes for Induction Chemotherapy

Toxicity is a major issue of IC, with up to 6% toxic deaths (9). Febrile neutropenia occurs in 11% (10) of cases and prophylactic granulocyte-colony stimulating factors (G-CSFs) are recommended by several institutions. Before the first administration of TPF, screening for dihydropyrimidine dehydrogenase deficiency is advisable. It can be partial (around 3–5% of individuals) or complete (0.2% of individuals) (21). Patients have to be managed by experienced oncologists familiar with supportive care.

On another note, IC could compromise the completion of subsequent concomitant CRT regimen. A Spanish phase III trial (11) comparing concomitant CRT preceded or not by IC-TPF showed a significant decrease of concomitant CRT completion rate: only 49.4% of patients were able to complete the three cycles of concurrent cisplatin 100 mg/m^2^ after IC, vs. 80.5% of patients treated exclusively with concomitant CRT.

The research is still ongoing to find fewer toxic schemes for IC.

It was shown that the modified TPF is less toxic than the conventional TPF in gastric cancers (22). A retrospective multicentric analysis which looked at 48 patients non-candidates for TPF (PS > 1, Age > 70, cardiac failure) who were treated with the modified TPF (docetaxel and cisplatin at 40 mg/m^2^ each on day 1, leucovorin 400 mg/m^2^, followed by a bolus of 5-FU at 400 mg/m^2^ and then 1,000 mg/m^2^/day, days 1–2, every 2 weeks) suggested similar efficacy with a response rate (RR) of 83%, and a better tolerance with 81% of patients who completed RTX. In this patient population 4% of toxicity-related mortality was reported (23). A randomized controlled trial comparing TPF to modified TPF in otherwise fit patients is currently ongoing.

Another promising regimen which combined carboplatin AUC2 and paclitaxel 135 mg/m^2^ weekly for 6 weeks (CT) was compared to TPF in a retrospective analysis (24). Fifty-three patients treated with CT had a better loco-regional control compared to the 90 patients treated with TPF in the same institution (80.5% for CT compared to 55.5% for TPF—HR 0.32, *p* = 0.0002) at 1 year. Progression free survival (PFS) was also higher with CT, and those results were confirmed in multivariate analysis. CT scheme increased neutropenia but TPF was associated with a worse renal toxicity.

Trying to eliminate 5-FU from the regimen, a large Korean multicenter phase II study (25) enrolled 92 patients to receive either three cycles of docetaxel and cisplatin with or without cetuximab (TP and TPE). Then, CRT was potentiated with both cetuximab and cisplatin in the TPE arm, whereas patients in the TP arm were potentiated with cisplatin alone. The addition of cetuximab did not increase the treatment efficacy. Three-year PFS rates were 70 and 56% for TPE and TP, respectively (*p* = 0.359), and 3-year OS rates were 88 and 74% (*p* = 0.313). TPE scheme proved to be more toxic and was associated with a greater need to reduce the dose.

### Attempts for Intensification of Induction Chemotherapy

Intensifying TPF without increasing toxicity remains a major issue and a goal of therapy that some trials have tried to achieve. A phase II trial with 50 patients potentiated TPF with weekly cetuximab infusions (C-TPF) for four cycles (26). Response rate was not better than TPF alone (86%, 95% CI 73–94%) but toxicity of this combination was highly increased, resulting in 24% febrile neutropenia, 20% grade 3–4 diarrhea, and 14% grade 3–4 mucositis. In a study from the EORTC group, 47 patients were treated with C-TPF, but the trial was stopped due to unacceptable toxicity (27). A North American trial with 30 patients replaced docetaxel by nab-placlitaxel (100 mg/m^2^) and suppressed the last day of 5-FU infusion in order to reduce toxicity and make the enhanced induction feasible. The following concomitant CRT was potentiated with cisplatin 100 mg/m^2^. Relapse occurred in only one of 30 patients, and 2-year PFS and OS were extremely promising (97 and 93%, respectively). Toxicity was moderate with 13% febrile neutropenia, 10% grade 3–4 diarrhea, and only 3% grade 3–4 mucositis (28).

The future step forward for IC will probably include immunotherapy. HNC is highly immunogenic (29), and prognosis has changed with the advent of immune checkpoint inhibitors (ICIs) (30, 31). Several trials aimed at improving IC efficacy with ICIs are recruiting patients. A phase I/II trial (32) planned to treat 55 patients with three cycles of TPF + pembrolizumab, a phase II trial (33) combining carboplatin, paclitaxel and nivolumab for three cycles and a phase I trial planned to treat 36 patients with three cycles of TPF + durvalumab (34) are ongoing.

## The Controversial Role of Induction Chemotherapy for Unresectable Non-Laryngeal Cancers

For fit patients with unresectable LAHNSCC, the standard of care remains concomitant CRT, combining RTX with cisplatin 100 mg/m^2^, except for patients candidates to laryngeal preservation, in whom IC followed by RTX is an alternative treatment option. Nonetheless, IC has several advantages and it must be considered.

To this date, there are five randomized controlled trials comparing IC + CRT vs. CRT alone for patients with high-risk LAHNSCC (Table 1).

**Table 1:** Randomized controlled essays comparing IC + concomitant CRT vs. concomitant CRT alone for HNSCC patients.

**Trial and scheme**	**Population characteristics**	**Outcomes**	**Bias and weaknesses**
**PARADIGM trial**			
- TPF + CRT (with carboplatin or docetaxel) vs. - CRT (with two cycles of cisplatin 100 mg/m^2^)	- 145 patients - N2 and N3 LAHNSCC Primary endpoint: OS	- No statistically significant difference in OS (HR, 1.09; 95% CI 0.59–2.03), in favor of IC	- Lack of power - Only 145/300 patients included - OS was higher than expected in the control arm
**TTCC trial**			
- TPF + CRT (three cycles of cisplatin 100 mg/m^2^) vs. - PF + CRT vs. CRT alone	- 439 patients - Stage III and IV HNSCC Primary endpoint: PFS	- No statistically significant differences in PFS (14.6 months in TPF + CRT; 14.3 months in PF + CRT; and 13.8 months in CRT alone)	- Many patients where ECOG 1 - Many high-volume tumors (T4)
**DeCIDE trial**			
- TPF + CRT (with fluorouracil, docetaxel, and hydroxyurea) vs. - CRT (with fluorouracil, docetaxel, and hydroxyurea)	- 285 patients - N2 and N3 HNSCC Primary endpoint: OS	- No statistically significant difference in OS (HR, 0.91; 95% CI, 0.59–1.41), in favor of IC - Strong trend in OS for high risk tumors (N2c and N3)	- Lack of power - Only 285/400 patients included - OS higher than expected
**GORTEC 2007-02 trial**			
- TPF + CRT (with cetuximab) vs. - CRT (three cycles of carboplatin + fluorouracil	- 370 patients - Stage III or IV HNSCC - N2b/c and N3 Primary endpoint: 2-y PFS	- No statistically significant differences in PFS and OS (HR, 1.10; 95% CI, 0.84–1.45) in favor of CRT - Higher distant free metastasis survival in TPF arm (HR, 0.62; 95% CI, 0.40–0.95)	- CRT scheme was not a standard (carboplatin + fluorouracil)
**GSTTC trial**			
First randomization: - TPF vs. -No IC Second randomization: - CRT potentiated with two cycles of cisplatin and fluorouracil vs. - CRT potentiated with cetuximab	- 421 patients - Stage III or IV HNSCC Primary endpoint: OS	- Increase of median PFS (29.7 months vs. 18.5, HR 0.72; 95% CI 0.56–0.93; *p* = 0.013) - Increase of median OS (HR 0.74; 95% CI 0.56–0.97; *p* = 0.031) - No benefit of IC for patient who received potentiation with cisplatin et fluorouracil	- CRT scheme was not a standard (cisplatin and fluorouracil or cetuximab) - This trial was not designed to investigate questions different from the efficacy comparison of IC vs. no-IC

The PARADIGM trial (10), a multicentric American study, did not show any difference in OS (HR 1.09; 95% CI 0.59–2.03), nor in PFS (HR 1.07; 95% CI 0.59–1.92) when comparing IC + CRT vs. CRT alone. The two treatment regimens produced similar outcomes, even considering high-risk patients (N2b-N3) in the subgroup analysis. However, this trial was burdened by several weaknesses such as the fact that the investigators randomized only 145 patients out of the 300 planned, and the 3-year follow-up was too short to highlight a difference between the two arms. Moreover, the control arm had survival rates higher than expected with a 3-year OS at 78% (95% CI, 66–86), so selection bias cannot be excluded.

The Spanish TTCC trial (11) was a larger phase III study that randomized 439 unresectable patients in three arms. CRT was potentiated with three cycles of cisplatin 100 mg/m^2^. In the control arm patients were treated exclusively with CRT, in the second arm CRT was preceded by IC-PF, and by IC-TPF in the third arm. Outcomes were similar in the intention-to-treat analysis (PFS 14.6, 14.3, and 13.8 months for TPF, PF, and CRT, respectively; OS 27, 27.2, and 26.6 months, respectively). After exclusion of the patients unable to complete the planned regimen (47/155 in the TPF group and 42/156 in the PF group) according to the per-protocol analysis, a non-significant trend for PFS was found in favor of induction over CRT (*p* = 0.083), while a significant benefit was reported for the comparison of TPF-CRT vs. CRT alone (HR, 0.719; 95% CI, 0.526–0.983; *p* = 0.03). As known, comparison in per-protocol analysis can lead to major bias by exclusion of unfit patients for IC and progressive disease during IC.

The second American trial, DeCIDE, limited to N2-N3 disease (12), compared CRT alone (hyperfractionated regimen delivering 0.15 Gray twice per day every other week, potentiated with docetaxel, 5-FU, and hydroxyurea) and CRT preceded by two cycles of IC-TPF. This American trial had similar flaws, with only 285/400 patients included, and OS was much higher than expected, similar in both arms (HR = 0.91; 95% CI 0.59–1.41). There was a strong trend for superior OS for high risk tumors (N2c and N3), but power was very low in this subgroup analysis with only 47 high-risk patients randomized.

The French GORTEC phase III trial (14) randomized 370 high-risk patients (N2b to N3) to receive either concomitant CRT (carboplatin 70 mg/m^2^ day 1 and 5-FU 600 mg/m^2^/day for four consecutive days every 3 week for three cycles) or IC with three cycles of TPF, followed by RTX and cetuximab in case of response or stable disease. PFS (HR = 0.95, *p* = 0.74) and OS (HR = 1.10; *p* = 0.48) were similar between the two arms. Interestingly, distant metastasis-free survival was higher in IC arm (HR = 0.62; *p* = 0.03). There were more grade 3–4 neutropenia and grade 3–4 fever with IC, but treatment completion was similar between the two arms.

The Italian GSTTC trial (13) was a factorial 2 × 2 study. Patients with unresectable LAHNSCC were randomized to receive concomitant treatment alone (RTX with two cycles of cisplatin and 5-FU—Arm 1a, or RTX with cetuximab—Arm 1b) or three cycles of IC-TPF followed by RTX with two cycles of cisplatin and 5-FU (Arm 2a) or followed by RTX with cetuximab (Arm 2b). Data about the two IC arms and the two exclusive concomitant CRT arms were pooled for statistical analysis. Outcomes were better in IC arms, with a significant increase of PFS (median 29.7 months vs. 18.5, HR 0.72; 95% CI 0.56–0.93; *p* = 0.013) and OS (HR 0.74; 95% CI 0.56–0.97; *p* = 0.031). Compliance to IC was very high as only 9 out of 208 patients did not receive the three planned TPF cycles. Furthermore, the concomitant treatments were not affected by induction. This trial showed that IC with TPF is safe and effective in a selected population. However, the potentiation scheme with two cycles of cisplatin and 5-FU is not a concurrent standard regimen, and the combination of RTX and cetuximab is not as efficient as cisplatin (35). In addition, subgroup analysis did not show any benefit for patients who received RTX with cisplatin and 5-FU after TPF.

## Induction Chemotherapy and Laryngeal/Hypopharyngeal Cancers

The only setting where IC has gained uniform consent as an effective treatment is resectable locally-advanced laryngeal and hypopharyngeal cancer. With the aim of organ preservation, patients less than stage T4a disease candidate to total laryngectomy, can be managed with sequential or concurrent CRT, with surgery as a secondary salvage option.

By the 1970's, conventionally fractionated RTX as a single modality has emerged as an alternative treatment to total laryngectomy for LASCC of the glottic and supraglottic larynx. Unfortunately, compared to surgery, RTX produced inferior rates of loco-regional control and survival (36–38).

From then on, total laryngectomy followed by adjuvant RTX has been considered the treatment of choice for patients with tumors not amenable to voice-sparing surgery. Such an aggressive approach improved both locoregional control and survival compared with RTX alone (38, 39), but these flattering results were achieved at the price of significant functional morbidity. The inevitable sequelae of total laryngectomy include a permanent tracheostomy and the loss of natural voice; furthermore, patients are at risk of developing rare complications such as alterations in swallowing ability or olfactory changes due to loss of nasal airflow, as well as taste disorders, that often result in psychological disturbances and social embarrassment (40–42).

The supremacy of surgery has been put to the test by the classical Veterans' Administration Laryngeal Cancer Study Group (VALCSG) trial, that successfully established the feasibility of larynx preservation without a negative impact on survival. Induction PF, followed by RTX in responders, was directly compared with total laryngectomy and demonstrated that a favorable response to IC could result in: (a) 64% rate of histologically confirmed complete responses after IC; (b) enhanced effect of RTX on the tumor; (c) preservation of a functioning larynx; (d) possible improvement in OS as a direct action of CHT on micrometastases.

In the trial, the 2-y OS was equivalent between the surgery arm and IC arm, but recurrence patterns were different, with a prevalence of distant failures in the former, and more frequent local failures in the latter (43).

The trial stressed the concept that a good response after two IC cycles can be associated with high rate of organ preservation and favorable survival (44).

In the RTOG 91-11, sequential CHT (with PF as induction) followed by RTX was compared to concomitant CRT and RTX alone in patients with locally-advanced laryngeal cancer (excluding T4 tumors with invasion through the full-thickness of the thyroid cartilage or base of tongue involvement), OS did not differ significantly between induction and concomitant arms, although the 10-year update analyses showed a trend toward a worse OS with concomitant CRT vs. sequential treatment (HR 1.25; 95% CI, 0.98–1.61; *p* = 0.08). DFS was significantly superior in the two arms combining CHT and RTX over RTX alone. Finally, the larynx preservation rate was significantly superior with CRT over IC followed by RTX (84% vs. 72%; *p* = 0.005) and over RTX alone (67%; *p* = 0.001). There was no difference among the three treatment groups with regard to speech (moderate or worse speech impairment) at 12 and 24 months of follow-up. Interestingly, at 1 year, 23% of the patients in the CRT group were able to swallow only soft foods or liquids, and 3% could not swallow at all, compared to 9% of the patients in the IC + RTX group who were able to swallow only soft foods or liquids, none of whom completely unable to swallow (*p* = 0.004). The results for the group treated with RTX alone were not significantly different from the other two groups (15 and 3%, respectively). At 2 years difficulty in swallowing was not different in the three groups (8).

Although no difference in late effects was detected, concomitant CRT caused more deaths not attributed to cancer or treatment (30.8% vs. 20.8% with IC) (9).

In the pre-TPF era, the EORTC (Head and Neck Cooperative Group) evaluated IC-PF followed by RTX vs. total laryngectomy in patients with advanced hypopharyngeal and aryepiglottic fold. If a partial response (PR) after two or three cycles of chemotherapy was obtained, RTX was administered (experimental arm). The primary endpoint was OS in terms of non-inferiority, compared to immediate surgery. As the median OS was 25 months in the immediate-surgery arm and 44 months in the IC arm (HR 0.86; log-rank test, *p* = 0.006, significantly less than the pre-definite < 1.43), the two treatments were considered to be equivalent. The 3- and 5-year estimates of retaining a functional larynx in patients treated in the IC arm were 42% (95% CI: 31–53%) and 35% (95% CI: 22–48%), respectively (45).

These results were confirmed by the long-term evaluation. The OS at 5 and 10 year was 32.6% (95% CI: 23.0–42.1%) and 13.8% (95% CI: 6.1–21.6%) in the surgery arm vs. 38.0% (95% CI: 28.4–47.6%) and 13.1% (95% CI: 5.6–20.6%) in the experimental arm. At 5 years, 59.5% of living patients in the CHT arm retained a normal larynx (46).

Subsequently, adding docetaxel to PF as IC demonstrated even more efficacy in terms of larynx preservation (47). The GORTEC 2000.01 phase III trial compared TPF vs. PF as IC in 213 patients with locally advanced larynx and hypopharynx carcinoma. The triplet combination led to an ORR of 80.0% (41.8% CR and 38.2% PR) in the TPF group and 59.2% (30.1% CR and 29.1% PR) in the PF group (difference = 20.8%; *p* = 0.002). Larynx preservation rate was 70.3% following TPF and 57.5% after the PF regimen (difference = 12.8%; *p* = 0.03). No significant difference was registered for OS between the 2 arms (60% each).

Following the results of Bonner trial (48), demonstrating that the combination of RTX and cetuximab was superior to RTX alone in loco-regional control and OS for patients with LASCCHN, the TREMPLIN trial was designed to evaluate the efficacy of TPF IC followed by conventional RTX with concurrent cisplatin or concurrent cetuximab as organ preservation strategies in laryngeal cancer. Patients who developed a tumor response ≥ 50% after three cycles of TPF were randomized to receive CRT or bioradiotherapy (BRT). No significant difference in terms of larynx preservation (95% vs. 93%), larynx function preservation (87% vs. 82%), and 18-months OS (92% vs. 89%) were observed. At a median follow-up of 36 months OS was 75% (95% CI: 62–85%) and 73% (95% CI: 60–84%) in the cisplatin arm and cetuximab arm, respectively (7).

Continuing to explore the role of cetuximab as a part of IC and BRT in locally advanced laryngeal cancer, Mesia et al. treated a total of 93 patients with induction TPF. After induction, 40% of the patients showed a complete response, 41% a partial response and 9% showed stabilization. Seventy-three patients (78%) received subsequent BRT. With a median follow-up of 53.7 months, the 3-y actuarial survival with a functional larynx, the laryngectomy-free survival (LFS) and the OS were 70% [95% confidence interval (CI) 60–79%]; 72% (95% CI 61–81%), and 78% (95% CI: 63–82%), respectively. These excellent results deserve further confirmation in a prospective phase III trial (49).

Unfortunately, data from a German multicenter randomized phase II larynx organ preservation (LOP) trial (DeLOS-II) did not confirm the hypothesis that cetuximab added both to IC-TPF and to RTX improves LFS in locally advanced laryngeal/hypopharyngeal cancer. After the randomization of 180 patients, 5-FU was omitted from IC because of 4 therapy-related deaths among the first 64 randomized patients. The primary objective (24 months LFS above 35%) was equally met by IC and RTX with or without cetuximab. The 24 months OS rates were 69.3 and 68.2%, respectively. No superiority of IC with TPF/TP and cetuximab was demonstrated regarding LFS and OS at 24 months (50).

Currently, TPF remains the gold standard as IC in laryngeal/hypopharyngeal cancer.

## Toxicity

### Acute Toxicity of IC

Hematological and renal toxicity are the main adverse events of induction TPF.

Since 2007, different TPF regimens have been developed with different single drug doses (3, 4, 13, 47, 51). The main dose differences in the developed TPF regimens concerned cisplatin and 5FU, while docetaxel at 75 mg/m^2^ on day 1 was administered in all trials. In addition, different number of cycles and different supportive procedures for the prophylaxis of high-grade hematological toxicity and infections were used. These differences also concerned the most recent phase III trials exploring the role of sequential treatment (i.e., induction TPF followed by concomitant treatment) as treatment intensification strategy (10–14).

Because the TPF regimens, with different cisplatin and fluorouracil doses, were developed independently of each other and no phase III trials comparing the different schedules exist, since their activity seem to be comparable, they can all be considered adequate as IC.

Despite differences in doses and number of cycles administered, it clearly appears that induction TPF is burdened by high-grade hematological toxicity, mainly leukopenia, neutropenia and febrile neutropenia (Table 2). For these reasons, prophylactic antibiotic administration (e.g., ciprofloxacin) is suggested after each cycle of TPF to decrease the risk of infections, while the use of G-CSF as primary prophylaxis is less established. High grade neutropenia and febrile neutropenia ranged from 19% to 36% and from 11% to 23%, respectively, in phase III trials exploring the role of IC-TPF as treatment intensification strategy. High grade thrombocytopenia and anemia were around 1–3% in all trials.

**Table 2:** G>3 Adverse Events of induction TPF in the Phase III trials comparing concomitant treatment vs. sequential treatment.

	**PARADIGM**	**DeCIDE**	**TTCC trial**	**GSTTC trial**	**GORTEC 2007.02**
Treatment arms	CDDP/RT vs. TPF -> T//RT or Cb/RT	DHF->RT vs. TPF-> DHF->RT	CDDP/RT vs. TPF -> CDDP/RT vs. PF-> CDDP/RT	PF/RT or CET/RT vs. TPF-> PF/RT or CET/RT	CbF/RT vs. TPF->CET/RT
Patients	145	280	439	414	370
TPF regimen	T 75 mg/m^2^ d1 P 100 mg/m^2^ d1 F1000 mg/m^2^/d, d1->4 c.i.	T 75 mg/m^2^ d1 P 75 mg/m^2^ d1 F 750 mg/m^2^/d, d1->5 c.i.	T 75 mg/m^2^ d1 P 75 mg/m^2^ d1 F 750 mg/m^2^/d, d1->5 c.i.	T 75 mg/m^2^ d1 P 80 mg/m^2^ d1 F 800 mg/m^2^/d, d1->4 c.i.	T 75 mg/m^2^ d1 P 75 mg/m^2^ d1 F 750 mg/m^2^ d1, d1->5 c.i.
TPF cycles	3	2	3	3	3
ATB prophylaxis	No	No	Yes	Yes	Yes
Primary prophylactic G-CSF	Yes	Yes	Yes°	No	Yes
Neutropenia	nr	36%	19%	27.5%	26%
FN	23%^*^	11%	17%	11%	17%
Anemia	nr	0.7%	2.7%	2.5%	nr
Platelets	nr	2.9%	3.3%	1%	nr
Stomatitis					
N/V					
Diarrhea					
Early death	1.4%	2.9%	5.2%	1%	6.6%

*CDDP, cisplatin; T, docetaxel; 5FU, 5-fluorouracil; Cb, carboplatin; PF, cisplatin plus 5Fluorouracil; DHF, docetaxel/hydrossiurea/5-Fluorouracile; CET/RT, cetuximab/RT; CbF, carboplatin plus 5Fluorouracil; ATB, antibiotics; FN, febrile neutropenia; c.i, continuous infusion*.

° *after protocol amendment*.

* *cumulative data for IC phase +concomitant phase*.

Patients candidate to induction TPF should be accurately selected for any organ dysfunction in relation to the potential toxicity expected from each drug, with regard to renal, neurological and cardiac dysfunctions. Moreover, pre-existing comorbidities as cirrhosis, uncontrolled diabetes, and neurological impairment could be a contraindication to induction TPF. For these reasons, all patients eligible for IC have to be accurately selected for comorbidities.

The main concern of the IC approach is the proportion of patients not able to start or to complete the subsequent concomitant treatment due to toxic events; however, it is difficult to draw definitive conclusions in this regard, because compliance and toxicity data are differently reported. In the phase III trials comparing concomitant vs. sequential treatment (Table 3) the rate of patients not receiving concomitant treatment after IC for any reason (including progressive disease, toxicity, early deaths for any causes, refusal) ranged from 8.8% to 10% in three trials [DeCIDE (10), PARADIGM (12), and GSTTC (13)] while higher rates were reported in the GORTEC 2007.02 study (14) (16.5%) and in the TTCC (11) trials (26%: 30.7% in the TPF arm and 22.4% in the PF arm) (Table 3). The analyses restricted to toxicity as a cause for not-starting concomitant treatment after IC was reported in only two trials: in the GSTTC trial (13) it was 2.4% while in the TTCC study (11) it was 12.2% (11.7% in the TPF arm and 12.8% in the PF arm). Different proportions of early deaths after TPF were also reported: 2.9% in the DeCIDE study (4 deaths, all treatment related) (10), 1.4% in the PARADIGM trial (1 patient, not treatment related) (12), 1% in the GSTTC trial (2 treatment related deaths) (13), and 3.8% in the TTCC trial (5.2% after TPF and 2.5% after PF) (11). The highest rate of early deaths of 6.6%, was observed in the GORTEC 2007.02 trial (14). This last result is difficult to explain because early deaths were mainly related to neutropenia associated infections, despite both prophylactic antibiotics and G-CSF were administered after each IC cycle.

**Table 3:** Proportion of patients not receiving concomitant treatment after induction TPF.

	**PARADIGM**	**DeCIDE**	**TTCC trial TPF arm**	**GSTTC** **trial**	**GORTEC 2007.02**
Never started RT: - PD -Early deaths -Toxicity -Others	10% nr 1.4^§^ nr nr	8.8% nr 2.9% nr nr	30.7% 2.6% 5.2% 11.7% 11.1%	10% 4% 1% 2.4% 2.6%	16.5% 5.5% 6.6% nr 4.4%

PD, progressive disease.

§ *total early deaths for IC + concomitant*.

Because of the possible serious toxicities resulting from induction TPF, this treatment modality should be administered to selected patients in controlled clinical setting by highly experienced medical oncologists.

### Acute Toxicity of Concomitant Treatments After IC

The National Comprehensive Cancer Network (NCCN) guidelines v1.201911 recognized two concurrent CHT regimens as “category 1:” high-dose cisplatin or carboplatin plus 5FU. No randomized trials comparing the effect of different platinum compounds (cisplatin vs. carboplatin) and of the platinum total dose have been performed.

Because of acute toxicity, literature data report suboptimal compliance to 3 cycles of concomitant CHT (both for high-dose cisplatin and for carboplatin + 5FU) with about 30% of patients unable to receive the three planned cycles (52, 53). Subset analyses seem to suggest that two cycles of concomitant CDDP monotherapy (200 mg/m^2^ total dose) may be as effective as three (52), but no randomized trials exist to confirm this hypothesis. Concurrent cetuximab or weekly carboplatin are alternative treatment options for patients unfit for cisplatin-based CHT.

Because of toxicities, intensive care and nutritional support are required during treatment to avoid interruptions and discontinuations. In-field toxicities are the most frequent acute adverse events reported. High-grade radiation-dermatitis and stomatitis occur in about 50–85% of the patients, depending on the RTX fractionation and on the concurrent regimen adopted (54).

In addition, systemic toxicity of concurrent drugs should be expected. If concurrent high-dose cisplatin is administered, gastrointestinal toxicity, neurotoxicity, renal impairment, and myelosuppression of any grade should be expected. Otherwise, if concurrent cetuximab is administered, specific toxicity of anti-EGFR agents such as acneiform rash and infusion reaction are frequently reported.

Two recent phase III trials, De-ESCALate trial (55) and RTOG 1016 non-inferiority trial (56), compared concomitant high-dose cisplatin (3 cycles concomitant to standard fractionated IMRT in De-ESCALate and 2 cycles concomitant to Accelerated IMRT in the RTOG 1016) vs. concomitant cetuximab in locally advanced HPV-positive oropharynx cancer. Both trials clearly showed higher OS and local control for concomitant cisplatin, while no significant difference in the proportion of high-grade toxicities (both acute and late) were reported. Nonetheless, significantly more serious adverse events were observed with cisplatin, mainly hospitalization due to gastrointestinal toxicity. The two trials confirmed a different spectrum of toxic events with higher incidence of systemic toxicities with cisplatin, as already reported in the randomized larynx preservation phase II TREMPLIN trial (7).

In the phase III trials exploring the role of sequential treatment, the analyses of toxicity and compliance to the concomitant phase of the treatment is clearly burdened by methodological limitations and by multiple variables. In some of the phase III trials comparing concomitant vs. sequential treatment, the cumulative toxicity data of the whole treatment (induction phase plus concomitant phase) were reported, while in others the toxic events were recorded separately for the different phases of treatment.

Moreover, only three trials (10, 11, 13) adopted the same concomitant treatment both in the sequential arm and in the control arm.

Concerning the in-field toxicities, in the DeCIDE trial (10) the incidence of high-grade stomatitis (47% vs. 51%, *p* = 0.48) and radiation-dermatitis (18% vs. 24%, *p* = 0.48) during concomitant treatment were similar, irrespective of IC, while high grade hematological toxicity was more frequently reported in patient receiving induction TPF. Conversely, G3-4 non-hematological toxicities were not worse in the IC arm and 98% of the patients completed the concomitant treatment.

In the GSTTC trial (13) high-grade in-field toxicities were also similar in both induction and concomitant arms. During the concomitant phase of the treatment, the incidence of G3-4 in-field mucositis and radiation-dermatitis (34.5% vs. 41% and 14% vs. 15%, respectively) were not increased in patients receiving IC. G3-4 neutropenia was significantly higher, but negligible (4% vs. 1%, *p* = 0.03) in patients receiving induction TPF, conversely, G3-4 non-hematological toxicities were not worse in the IC arm. Completion of the concomitant treatment phase was similar, too. The proportion of patients able to complete the planned RTX was 93% in both arms and the completion of the planned concomitant systemic treatment was 88% in the control arm and 85% in the sequential arm.

The TTCC trial (11) was the only trial in which 3 cycles of concurrent high-dose cisplatin were administered both in the concomitant arm and in the two induction arms (TPF or PF). Grade 3–4 stomatitis was more frequently reported in the IC arms (49% in the TPF arm and 50% in the PF arm vs. 33% in the concomitant CRT arm) while the incidence of radiation-dermatitis was not reported.

Renal dysfunction related to cisplatin is another issue that could affect the completion of the concomitant treatment, especially in the sequential strategy if concurrent high-dose cisplatin is used. In the TTCC trial the planned cumulative cisplatin total dose was >500 mg/ m^2^ in both sequential arms (IC + concomitant) vs. 300 mg/m^2^ in the control arm. G3-4 renal dysfunction was reported in a significant number of patients in all the arms: 8.4% in the TPF arm, 3.1% in the PF arm, and 5.1% in the concomitant CRT arm. About 31% of patients received <3 concomitant cisplatin cycles (40.5% in the TPF arm, 34% in the PF arm, and 19.5% in the concomitant CRT arm). Fifteen percent of the patients in the IC arms discontinued concomitant treatment (17% in the TPF arm and 13% in the PF arm), while 18% treatment discontinuation was observed in the control arm. These data confirm the sub-optimal compliance to 3 cycles of concomitant high-dose cisplatin, especially in patients receiving cisplatin-based IC.

The best compliance to concomitant treatment reported in the Italian trial (13) vs. the Spanish trial (11) might be attributable to the schedule and doses of TPF and to the concomitant regimen adopted (2 cycles of concurrent cisplatin plus 5FU). It should be noted that in the GSTTC trial the cisplatin total dose in the sequential arm was 400 mg/m^2^ vs. >500 mg/m^2^ in the Spanish trial.

Because concomitant treatments are burdened by high-grade adverse events, it is well-established that supportive measures should be instituted early, independently from the concomitant regimen adopted, with the aim of reducing treatment-related side effects and minimizing delays and treatment modifications.

### Late Toxicity of CRT

Data regarding late toxicity of concomitant treatments have not been clarified due to lack of uniform collected data. Most common reported complications include long-term dependence from feeding tube, and xerostomia. Machtay et al. analyzed factors associated with late dysphagia according to 3 RTOG prospective trials (91-11; 97-03 and 99-14). According to the Authors, 43% of assessable patients reported severe late toxicity, mainly pharyngeal or laryngeal dysfunction. A negative correlation was found between the rate of late toxicity and older age, advanced T stage, larynx/hypopharynx primary site, and neck dissection after CRT (57). The adoption of IMRT technique reduced the rate of late severe toxicity, including dysphagia, due to the possibility of sparing the constrictor muscles from the high dose fields. Feng et al. demonstrated that doses higher than 60 Gy to pharynx constrictor muscles, glottic larynx, and supra-glottic larynx were associated with increased dysphagia (58), while Dornfeld et al. observed a cut-off dose at 50 Gy (59).

Even for salivary gland functions, IMRT has been demonstrated to be advantageous in terms of toxicity reduction. The PARSPORT trial included 94 oropharynx or hypopharynx cancer patients, randomized to receive conventional RTX or IMRT. At 24-month evaluation, patients randomized to IMRT had a reduced incidence of xerostomia (29 vs. 83%, *p* < 0.0001) (60).

When analyzing late toxicities reported in trials investigating the role of IC, a complete review of side effects was reported by the Pittsburgh Cancer Institute (6). The most common complication in the 39 enrolled patients was G-tube placement, reported in 51% of the cases: in 5 patients the G-tube was placed before starting IC, in one patient before RTX with concomitant cisplatin and cetuximab (XPE), and in 14 patients during the XPE course. Other grade 3 or 4 side effects were rare: one patient developed laryngeal chondro-radionecrosis and another one laryngeal edema. Chronic grade 2 neuropathy were reported in two cases. In the TREMPLIN trial, late toxicity did not differ significantly between the two groups of treatment except for residual grade 1 renal dysfunction in patients receiving concurrent cisplatin. Xerostomia was the most frequently reported late adverse event with an incidence of 10.3 and 8.9% in the cisplatin and cetuximab arms, respectively. In addition, laryngoesophageal dysfunctions requiring laryngectomy, tracheotomy, and/or feeding tube use (8.6% vs. 9%) and subcutaneous fibrosis (7.0% vs. 2.0%) were reported (7). In the PARADIGM trial (10) the Authors did not separate acute from late toxicity, however no difference among induction vs. no induction arms were observed in terms of high-grade xerostomia (7% in both arms), neuropathy (0% vs. 3%) and PEG tube placement (79% vs. 85%).

## Conclusion

Beyond surgery, selected patients with LAHNSCC can be treated with RTX or a combination of RTX and CHT with the aim of sparing organ function. The role of IC before RTX or before CRT is still debated, as data on its efficacy are somehow confusing, but in laryngeal and hypopharyngeal cancer it is one of the recommended organ preservation strategy.

The standard CHT regimen for IC is TPF. Adding taxans to PF improves the response rate, the PFS and the OS rates. Moreover, the TPF regimen demonstrated a more favorable toxicity profile compared to PF and a better treatment completion rate.

Toxicity of IC remains a major issue with up to 6% mortality rate, but reduced intensity schemes are promising. Modified TPF schedule and the combination of paclitaxel-carboplatin are feasible and seem to be similarly effective. These alternative regimens remain to be validated through comparative trials.

Studies advocating the potentiation of TPF with another cytotoxic drug were inconclusive because of the high toxicity rates. However, several trials are now considering the addition of ICIs to TPF or concomitant CRT (61–66).

Together with chemoradiation, induction TPF followed by RTX or by CRT is a non-inferior choice in selected patients, with the potential advantage of improving the loco-regional and distant control. Further investigation in phase III trials is warranted to determine the optimal treatment after induction TPF.

## Author Contributions

DF contributed conception and design of the paper. DF, MGG, CF, CC, MG, and JF wrote sections of the manuscript. All authors contributed to manuscript revision, read and approved the submitted version.

### Conflict of Interest

The authors declare that the research was conducted in the absence of any commercial or financial relationships that could be construed as a potential conflict of interest.
